# The Inhibitory Effect of *Haloxylon salicornicum* on Contraction of the Mouse Uterus

**DOI:** 10.1155/2013/714075

**Published:** 2013-09-23

**Authors:** Nabila H. Saleem, Valerie A. Ferro, Ann M. Simpson, John Igoli, Alexander I. Gray, Robert M. Drummond

**Affiliations:** Strathclyde Institute of Pharmacy and Biomedical Sciences, University of Strathclyde, 161 Cathedral Street, Glasgow G4 0RE, UK

## Abstract

*Haloxylon salicornicum* (*H. salicornicum*) is a plant that is frequently taken as a tea by Bedouin women in Egypt who are experiencing difficulties during pregnancy, as well as to provide relief from dysmenorrhoea. Despite its medical use, there has been no detailed evaluation of the effect of this plant on uterine tissue. Therefore, the initial aim of this study was to determine whether *H. salicornicum* affected the contraction of the mouse uterus *in vitro*. The crude aqueous extract of *H. salicornicum* was found to inhibit the spontaneous contractions of the uterus, with the effect being rapid in onset and completely reversible upon washout. Subsequent purification of the plant extract resulted in the identification of synephrine and N-methyltyramine, both of which were found to have inhibitory effects on the spontaneous contractions of the uterus. The EC_50_ for the purified constituent identified as synephrine was 0.82 ± 0.24 **μ**g/mL. The inhibitory activity of crude *H. salicornicum*, as well as the isolated constituents, could be prevented by pretreatment of the uterus with the **β**-adrenoceptor antagonist propranolol. In conclusion, the use of *H. salicornicum* during preterm labour appears to be justified, and its pharmacologic effect is consistent with it acting as a **β**-adrenoceptor agonist.

## 1. Introduction

Regulation of the contractile status of the uterus is of fundamental importance for a successful outcome in pregnancy. Throughout gestation the uterus has to remain relatively quiescent in order to prevent abortion, while at the end of the gestation period it has to produce the regular forceful uterine contractions that are required to bring about parturition [1–3]. If the uterus starts to contract too early, this will lead to premature birth, which is the most important single determinant of adverse neonatal outcome, both in terms of survival and quality of life. In 2010, it was estimated that worldwide, 14.9 million babies (11.1% of all live births) were born preterm (<37 weeks of completed gestation) [4]. There is considerable variation between different regions throughout the world; for instance, the estimated preterm birth rate in developed regions is 8.6% while in sub-Saharan Africa it is 12.3% [4]. Furthermore, in sub-Saharan Africa 28% of neonatal deaths are due entirely to their premature delivery [5], compared with 4-5% of the neonatal deaths in developed countries. Unfortunately, maternal deaths during pregnancy are also significantly higher in sub-Saharan regions than they are in developed regions [6].

For women who are in preterm labour, a wide variety of pharmacological agents have been utilised to suppress uterine contractions (so-called tocolytics) [7]. Tocolytics that are in current clinical use include *β*_2_ adrenergic receptor agonists, calcium channel blockers, magnesium sulphate (MgSO_4_), nitrates, prostaglandin synthetase inhibitors, and oxytocin receptor antagonists [8, 9]. These tocolytics all have different mechanisms of action and a number of adverse effects associated with them, which need to be taken into account when considering which drug to use. While there is no standard first line drug that is universally used, a recent meta-analysis study has indicated that calcium channel blockers and prostaglandin inhibitors have the highest probability of delaying delivery and improving neonatal outcomes [9]. Magnesium sulphate remains popular as a tocolytic in the USA and some other parts of the world; however, it is rarely used for this indication in the UK.

In developing countries, there is often a reliance on traditional medicines to treat medical conditions. Indeed, a number of clinically effective tocolytics have been derived from plant sources [10], and herbals are often used in the treatment of painful menstrual cramps that are of uterine origin (dysmenorrhea) [11]. The Bedouin women of Egypt rely mainly on traditional medicine, and one of the herbals that they are known to use during pregnancy and in the relief of dysmenorrhea is the plant *Haloxylon salicornicum *(Moq.) Bunge ex Boiss* (H. salicornicum)*. During interviews with Bedouin women in St. Catherines, Egypt, it was revealed that when used for these purposes, the plant is taken as a tea 3-4 times per day [12].

*H. salicornicum* belongs to the Chenopodiaceae family, and it is widely distributed in Northern Africa and Asia, in both temperate and tropical regions [13, 14], where it grows in sandy and stony desert areas as a diffuse shrub [15]. *H. salicornicum* is known to contain a number of alkaloids, including piperidine alkaloid [16], haloxynine, and haloxine [17]. In addition it also contains pyranones [18], tannins, saponins, and a number of glycosides [14].

Although *H. salicornicum* is used by Bedouin women as a herbal medicine, to alleviate a number of obstetric and gynaecological problems, there has been no systematic study into its actual pharmacology [12]. Therefore, the initial aim of this study was to establish whether the crude extract of *H. salicornicum*, obtained from the same parts of the plant that are used by Bedouin women, had any effect on the contractility of the mouse uterus. Thereafter, subsequent investigations were directed towards identifying the active constituent(s) in *H. salicornicum* as well as their mechanism of action on the uterus.

## 2. Materials and Methods

### 2.1. Extraction and Purification

#### 2.1.1. Plant Material

*H. salicornicum *was collected from Saint Catherine in the South Sinai peninsula, Egypt, and dried by a traditional technique that is utilised by Bedouin women. In particular, the aerial parts of the plants were collected and placed on rocks in shaded areas to dry. Two-three days later, the dried plants were collected and ground to a powder using a mortar and pestle. Thereafter, the plant material (320 g) was extracted using a Soxhlet extractor with a methanol and water mixture (1 : 1 v/v). After evaporation of the methanol, the extract was freeze dried.

#### 2.1.2. Phytochemical Screening

The freeze dried extract (18 g) was redissolved in 100 mL water and was partitioned by adding 250 mL chloroform. The organic phase was dried over anhydrous sodium sulphate, filtered, and evaporated to dryness at 40°C under vacuum using a rotary evaporator (Büchi R-205, Oldham, UK). The dried extract was then dissolved in methanol and applied to a Sephadex LH 20 column with bead size 25–100 *μ*m (Sigma-Aldrich, UK) and eluted with methanol to obtain 30 fractions, which were 100 mL each. Fractions 1–15 were identical on thin layer chromatography (TLC) with methanol-ethyl acetate (20 : 80 v/v), and were therefore combined to obtain subfraction (A). Similarly, fractions 16–30 were combined to obtain subfraction (B). Subfraction (B) was then subjected to C-18 silica gel column chromatography (Phenomenex, Macclesfield, UK) and elution with water/methanol to obtain 32 fractions. Subfraction (C) was made up of collected fractions 1–16, while subfraction (D) was made up of fractions 17–32, after TLC analysis had indicated that these collected fractions were identical. Approximately 500 mg of dried subfraction (D) was chromatographed by medium pressure liquid chromatography (MPLC) utilising a gradient of EtOAc and MeOH from 100 : 0 to 90 : 10 at a flow rate of 20 mL/min for 50 min. This yielded 200 fractions of 5 mL each. The eluted fractions, which were deemed to be identical by TLC analysis, were pooled to yield HS1, HS2, HS3, and HS4. NMR studies (1D and 2D) as well as liquid chromatography-mass spectrometry (LC-MS) were carried out to elucidate the structures of the compounds and fractions that were isolated.

#### 2.1.3. Animals

Adult virgin female C57BL/6 mice (20–30 g) were used in this study. The mice were humanely killed by cervical dislocation, and all studies were performed in accordance with the guidelines and principles for the care and use of laboratory animals at the University of Strathclyde.

#### 2.1.4. Tissue Preparation and Organ Bath

Following a longitudinal midline incision, the uterus was carefully removed and immediately immersed in cold physiological saline solution (PSS) comprising (mM: NaCl 118.4, KCl 4.7, MgSO_4_ 1.2, glucose 11, CaCl_2_ 2.5, NaHCO_3_ 25, KH_2_PO_4_ 1.2, pH 7.4 and gassed with 95% O_2_-5% CO_2_). The uterus was then placed in a Sylgard coated dissecting dish containing cold PSS, and the right and left uterine horns were carefully separated, with the aid of a dissecting microscope. The right and left uterine horns were mounted vertically in 10 mL organ baths, one end being fixed to a stainless steel gas bubbler and the other end connected to a force displacement transducer (Grass FT03, Astro-Med, Slough, UK) using silk thread. The output from the transducer was amplified by a PowerLab 4/35 and the data recorded on a personal computer running LabChart v7 software (ADInstruments Ltd., Oxford, UK). A resting tension of 0.5 g was applied, and the uteri were allowed to contract spontaneously during an initial equilibration period of 30–60 min, before commencing any experimental protocol. All experiments were carried out at 37°C, and the PSS was continuously gassed with 95% O_2_-5% CO_2_.

### 2.2. Effect of the Crude Aqueous Extract of *H. salicornicum* and Its Isolated Constituents on Spontaneous Contractions of the Mouse Uterus

Following the initial equilibration period, either the crude aqueous extract or one of the *H. salicornicum* fractions was applied to the tissue. All substances were dissolved in 10% (v/v) aqueous DMSO and were applied either as a single addition followed by washout or in a cumulative manner (0.1–3 *μ*g/mL). Whilst 10 min intervals were allowed between subsequent applications when the additions were cumulative, the effect had normally reached steady state well within the 10 min period. In parallel control experiments, the effect of the vehicle (aqueous DMSO) on the contraction of the uterus was also examined.

### 2.3. Effect of Propranolol on the Inhibitory Action of *H. salicornicum* and Its Isolated Constituents

After obtaining inhibition of the spontaneous uterine contractions with either the crude aqueous extract of *H. salicornicum* or one of its isolated constituents, the tissue was washed with fresh PSS, and the spontaneous contractions were allowed to recover. The nonselective *β*-antagonist propranolol (20 *μ*M) was then applied to the tissue, and following a 10 min equilibration period, the effect of crude *H. salicornicum* (3 *μ*g/mL) or its isolated constituents were then re-examined in the continued presence of propranolol.

### 2.4. Effect of *H. salicornicum* and Its Isolated Constituents on KCl-Induced Contraction of the Mouse Uterus

The uterus was initially contracted by the application of KCl (60 mM), and after the contractile response was stable, either the crude aqueous extract of *H. salicornicum* or one of its isolated constituents was added to the tissue in a cumulative manner (0.1–3 *μ*g/mL). In some experiments, *H. salicornicum* or one of its isolated constituents was added as a single concentration (3 *μ*g/mL).

### 2.5. Chemicals

Propranolol and (+/−) synephrine (both from Sigma-Aldrich, Gillingham, UK) were each prepared as 100 mM stock solutions in Milli-Q water, and subsequent dilutions were made using PSS. Dimethyl sulphoxide (DMSO) and methanol (HPLC grade) were obtained from Sigma-Aldrich, UK. All other reagents used were from BDH (VWR, Lutterworth, UK) and were of AnalaR grade.

### 2.6. Data Analysis

Measurement of the spontaneous contractile activity was carried out by calculating the integral (area under the curve) of the contraction-time record using the LabChart software. The integrated contractile response for the 6 min period preceding the addition of any substance served as a control whilst a similar 6 min period during application of the substance was used to examine its effect. The data was then expressed as a percentage of the integrated contractile response prior to any substance being applied (i.e., percentage of the control). The frequency and amplitude of the contractile responses during the period that was integrated were also determined and expressed as a percentage of the control prior to any addition.

The data are shown as the mean ± s.e.m., and *n* is the number of uteri from different animals. EC_50_ values for the isolated constituents were calculated by fitting the data for the contractile integral to the Hill Equation. One-way analysis of variance with Dunnett's post hoc test was used to compare the treatment to the control group (GraphPad Prism v4), and *P* < 0.05 was considered to be statistically significant.

## 3. Results

### 3.1. Isolation and Identification of the Active Compounds in *H. salicornicum*

Four compounds HS1, HS2, HS3, and HS4 were isolated and characterized from subfraction (D) of *H. salicornicum*. These compounds were identified as synephrine (HS1) and a mixture of synephrine and N-methyltyramine (HS2) by NMR spectroscopic, LC-MS analysis, and comparison with previously published data [19]. HS2, which was actually a mixture of N-methyltyramine and synephrine, had a molar ratio of 1.6 : 1 (based upon the integrals of the aromatic AA′BB′ signals with the deshielded multiplets at *δ*_H_ 7.19 and *δ*_H_ 7.31 or the N-methyls at *δ*_H_ 2.69 and *δ*_H_ 2.77, resp.). The other purified constituents were identified as piperidine (HS3) and allantoin (HS4) [20]. Neither HS3 nor HS4 was found to have any effect on mouse uterine contractility and was therefore not investigated any further during this study.

### 3.2. Effect of the Crude Extract of *H. salicornicum* on Spontaneous Contractions of the Mouse Uterus

**Figure 1:**
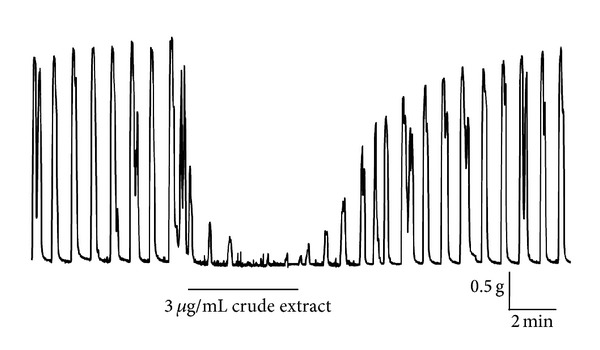
Effect of the crude extract of *H. salicornicum *on spontaneous contractions of the mouse uterus. Representative recording showing the effect of the crude aqueous extract of *H. salicornicum* (3 *μ*g/mL) on spontaneous contractions of the mouse uterus. The inhibitory effect of the extract was rapid in onset and could be completely reversed upon washout.

The crude aqueous extract of *H. salicornicum* decreased the spontaneous contractions of the mouse uterus in a concentration-dependent manner. At the highest concentration examined (3 *μ*g/mL), the crude extract almost completely abolished the contractions (Figure 1). The inhibitory effect of the crude extract was evident immediately upon application and was maintained as long as the extract was present in the organ bath. Upon washout of the extract, the inhibitory effect was readily reversed, and the effect could be consistently reproduced on subsequent readdition of the extract. The vehicle alone (aqueous DMSO), which was used in this and subsequent studies involving the isolated constituents, had no effect on the spontaneous contractions of the mouse uterus.

### 3.3. Effect of HS1 on Spontaneous Contractions of the Mouse Uterus

**Figure 2:**
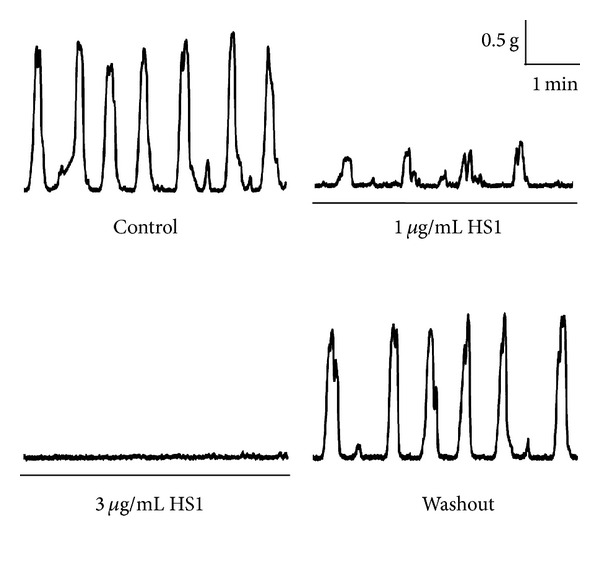
Effect of HS1 on spontaneous contractions of the mouse uterus. Representative recordings showing the effect of HS1 (1 and 3 *μ*g/mL) on spontaneous contractions of the mouse uterus. HS1 was one of the constituents purified from *H. salicornicum *and found to have an inhibitory effect on uterine contractions. All recordings are from the same mouse uterus and are shown in discontinuous form for clarity.

**Figure 3:**
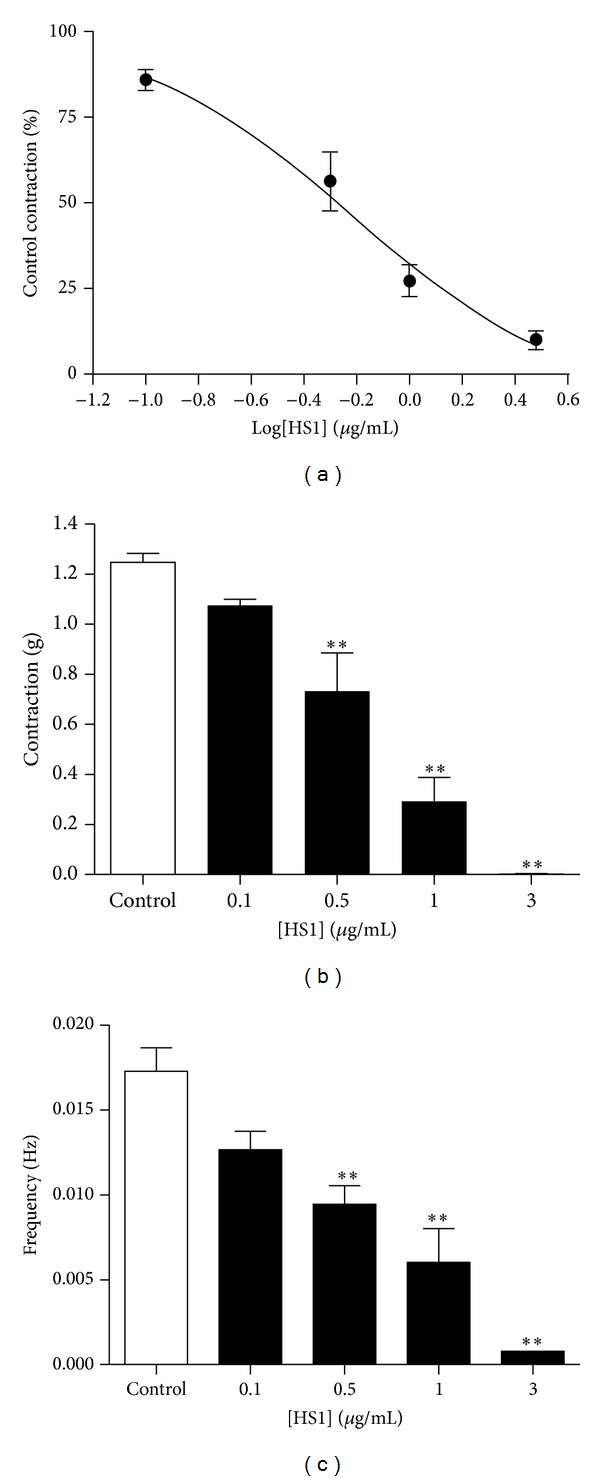
Effect of HS1 on spontaneous contractions of the mouse uterus. (a) Concentration response curve examining the effect of HS1 on spontaneous contractions of the mouse uterus. The data represents the integral of the contraction-time record and is expressed as a percentage of the control response in the absence of HS1. The curve has been fitted to the Hill Equation. The effect of HS1 on the amplitude (b) and frequency (c) of the spontaneous contractions is also shown. The data is presented as mean ± s.e.m and *n* = 5. ** indicates that *P* < 0.01 HS1 treatment versus control.

The purified constituent of *H. salicornicum,* HS1 (0.1–3 *μ*g/mL), inhibited the spontaneous contraction of the mouse uterus in a concentration-dependent manner (Figure 2). As assessed by the integral of the contraction-time record, 0.5 *μ*g/mL HS1 significantly decreased the contractile response by 44 ± 9% (*n* = 5) (*P* < 0.01), when compared to control (Figure 3(a)). This decrease in spontaneous contractility was due to a significant decrease in both frequency and amplitude of the contractions, by 43 ± 9% (*P* < 0.01) and by 42 ± 11% (*P* < 0.01), respectively (Figures 3(b) and 3(c)). Increasing the concentration of HS1 to 1 *μ*g/mL resulted in the contractile response being reduced by 73 ± 5% of the control (*P* < 0.01), with further decreases in the frequency and amplitude as well. HS1 (1 *μ*g/mL) decreased the frequency of the spontaneous contractions by 62 ± 14% (*P* < 0.01) and the amplitude by 77 ± 8% (*P* < 0.01). The highest concentration of HS1 examined (3 *μ*g/mL) completely abolished the spontaneous contractions in three out of the five tissue preparations. The EC_50_ for HS1 was 0.82 ± 0.24 *μ*g/mL (Figure 3), and its inhibitory effect was fully reversed within minutes when the tissue was washed with fresh PSS.

### 3.4. Effect of Synephrine on Spontaneous Contractions of the Mouse Uterus

**Figure 4:**
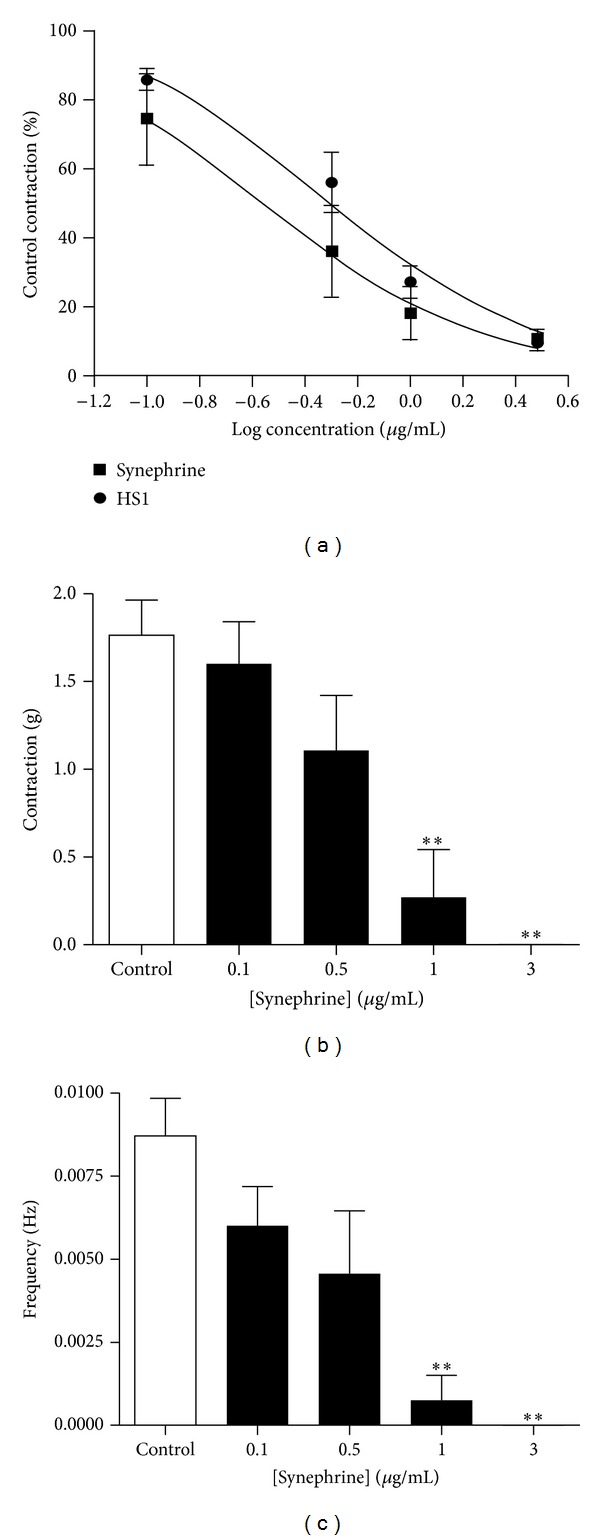
Effect of synephrine on spontaneous contractions of the mouse uterus. (a) Concentration response curve examining the effect of synephrine on spontaneous contractions of the mouse uterus. The data represents the integral of the contraction-time record and is expressed as a percentage of the control response in the absence of synephrine. The curve has been fitted to the Hill Equation and the HS1 data from Figure 3 is included for comparison. The effect of synephrine on the amplitude (b) and frequency (c) of the spontaneous contractions is also shown. The data is presented as mean ± s.e.m and *n* = 4. ** indicates *P* < 0.01 synephrine treatment versus control.

Since HS1 was identified by NMR spectroscopy as synephrine, the effect of commercially supplied synephrine on uterine contractility was examined to confirm that these two substances had similar effects. Synephrine also inhibited the spontaneous contractions of the mouse uterus in a concentration-dependent manner, and the EC_50_ was 0.34 ± 0.16 *μ*g/mL (*n* = 4, Figure 4(a)). In the presence of 0.5 *μ*g/mL synephrine, the integral of the contraction was significantly reduced by 65 ± 11% of the control (*P* < 0.01), with the amplitude and frequency decreasing by 38 ± 14% and 49 ± 1%, respectively (Figures 4(b) and 4(c)). The spontaneous contractions were abolished in three out of four preparations with 1 *μ*g/mL synephrine and in all preparations when the concentration was increased to 3 *μ*g/mL.

### 3.5. Effect of HS2 on Spontaneous Contractions of the Mouse Uterus

**Figure 5:**
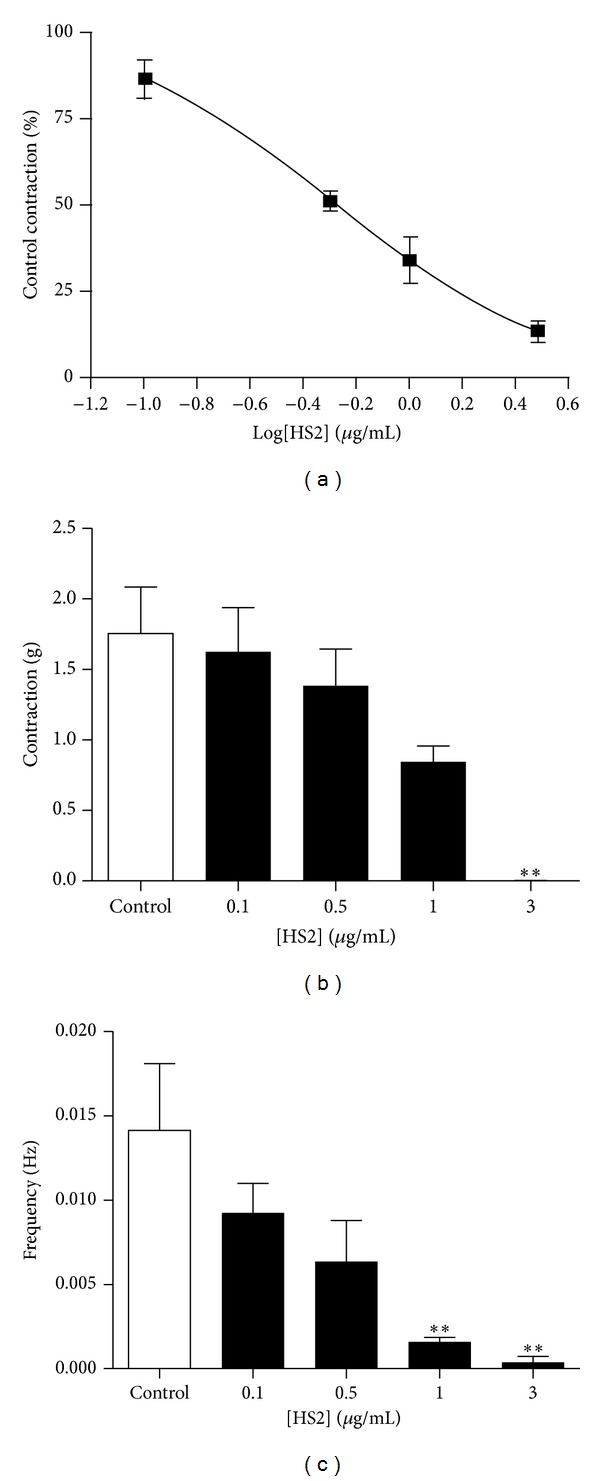
Effect of HS2 on spontaneous contractions of the mouse uterus. (a) Concentration response curve examining the effect of HS2 on spontaneous contractions of the mouse uterus. The data represents the integral of the contraction-time record and is expressed as a percentage of the control response in the absence of HS2. The curve has been fitted to the Hill Equation. The effect of HS2 on the amplitude (b) and frequency (c) of the spontaneous contractions is also shown. The data is presented as mean ± s.e.m and *n* = 3. ** indicates that *P* < 0.01 HS2 treatment versus control.

Application of HS2 (0.1–3 *μ*g/mL) also inhibited spontaneous contractions of the mouse uterus in a concentration-dependent manner, yielding an EC_50_  0.62 ± 0.31 *μ*g/mL (*n* = 3, Figure 5(a)). HS2 (1 *μ*g/mL) significantly inhibited the spontaneous contractions of the uterus by 66 ± 7% (*P* < 0.01) when compared to the control. The same concentration of HS2 decreased both amplitude and frequency of the spontaneous contractions by 50 ± 7% and 86 ± 5% (*P* < 0.01), respectively (Figures 5(b) and 5(c)) Increasing to concentration of HS2 to 3 *μ*g/mL completely abolished the spontaneous contractions in two out of the three tissue preparations.

### 3.6. Effect of Propranolol on the Inhibitory Action of HS1, Synephrine, and HS2

**Figure 6:**
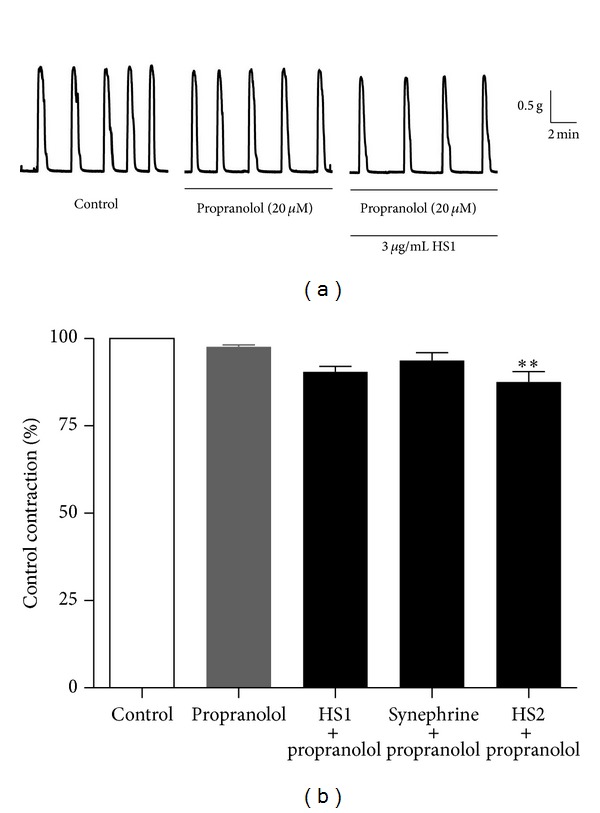
Effect of propranolol on the inhibitory action of HS1 and HS2. (a) Representative recording showing spontaneous contractions of the mouse uterus and how they are unaffected by the nonselective *β*-antagonist propranolol (20 *μ*M). In the presence of propranolol the inhibitory action of HS1 (3 *μ*g/mL) was largely prevented. All recordings are from the same mouse uterus. (b) Summary data showing how propranolol affects the inhibitory action of HS1, synephrine, and HS2 (all at 3 *μ*g/mL) on the mouse uterus. The data represents the integral of the contraction-time record and is expressed as a percentage of the control response in the absence of propranolol. The data is presented as mean ± s.e.m and *n* = 3. ** indicates that *P* < 0.01 for HS2 versus propranolol alone.

The nonselective *β*-adrenoceptor antagonist propranolol (20 *μ*M), by itself, was found to have no effect on the spontaneous contractility of the uterus. The integral of the contraction-time record was 98 ± 1% of the control obtained in the absence of propranolol (Figure 6). When HS1 (3 *μ*g/mL) was added in the presence of propranolol, the inhibitory effect normally seen was largely prevented. HS1 still caused a small reduction in contractility, by 10 ± 2% from the control; but this was not significantly different from the effect of propranolol alone. The inhibitory effect of the commercially obtained synephrine was also prevented by propranolol, with synephrine only reducing the spontaneous contractions by 6 ± 2%. Although HS2 (3 *μ*g/mL) was still capable of producing a small reduction in spontaneous contractility (by 12 ± 3% of the control) in the presence of propranolol, it was also much less than that previously observed in the absence of propranolol.

### 3.7. Effect of HS1 on Uterine Contractions Induced by Depolarization

**Figure 7:**
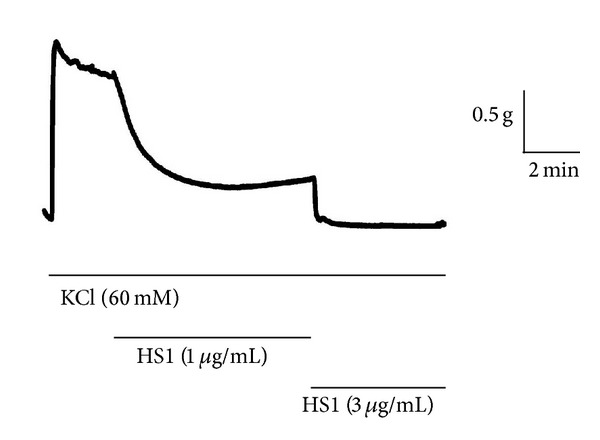
Effect of HS1 on KCl-induced contraction of the mouse uterus. Representative recording showing the inhibitory action of HS1 (1 and 3 *μ*g/mL) on the mouse uterus that had been precontracted with KCl (60 mM). This figure is representative of 3 similar independent experiments.

Application of KCl (60 mM) to the uterus produced a biphasic contraction, comprising an initial transient contraction followed by a relatively sustained plateau. When HS1 (1–3 *μ*g/mL) was applied during the sustained phase of the contraction, it caused a concentration-dependent relaxation, with complete relaxation to baseline tension being achieved with the highest concentration of HS1 examined (3 *μ*g/mL) (Figure 7).

## 4. Discussion

The crude extract of *H. salicornicum *was found to have an inhibitory effect on the spontaneous contractions of the mouse uterus, which was rapid in onset and fully reversible upon washout. Purification of the extracts, obtained from *H. salicornicum*, resulted in the identification of HS1 (synephrine) and HS2 (a mixture of synephrine and N-methyltyramine), which were subsequently shown to be responsible for the inhibitory effect of this plant on the contractions of the mouse uterus. Furthermore, the effects of HS1 and HS2 were concentration dependent and were mediated by the activation of *β*-adrenoceptors since their inhibitory action could be prevented by prior treatment of the uterus with propranolol.

It has long been known that stimulation of *β*-adrenoceptors is responsible for smooth muscle relaxation in many organs including the uterus [21, 22]. Studies on the responsiveness of the uterus to *β*-adrenoceptor agonists, as well as characterisation of myometrial *β*-adrenergic binding sites in several species (human, rat, and guinea pig), have indicated that *β*_2_ is the dominant subtype present in this tissue [23–25]. *β*_2_-adrenoceptor agonists are known to inhibit contraction of the uterus by stimulating the production of cAMP [26]. They are effective in delaying delivery for up to 48 hours and have no effect on perinatal mortality or morbidity [27]. At the end of pregnancy, the expression of *β*_2_-adrenoceptors in the uterus has been reported to either decrease [24] or remain unchanged [28]. Certainly in humans, as well as in rodents, the *β*-adrenoceptor signalling pathway desensitizes at the end of pregnancy, thereby contributing to the initiation of contractions at parturition [29, 30]. There is also a loss of responsiveness of the uterus to *β*-adrenoceptor agonists following long-term exposure to these drugs [31]. These factors are likely to underlie the reason why *β*-adrenoceptor agonists become less effective tocolytics at the end of pregnancy. The most widely reported adverse effects of therapeutic doses of *β*_2_-adrenoceptor agonists are skeletal muscle tremor [22] and cardiac effects [32]; which does cause some limitations in their clinical use as tocolytics. 

Phenolic amines, which include synephrine, are a class of secondary metabolites found in citrus fruits [33] and other edible plants that have been reported to have a wide range of health promoting activities [34, 35]. Synephrine is used as a sympathomimetic agent [35], and its structural isomer neosynephrine (phenylephrine) is used in the treatment of common colds. Recently, synephrine has received increasing attention as results from several studies, including human trials [36], have suggested that it has promise as an aid to weight management and obesity reduction [35]. Synephrine is also known to be capable of causing vasoconstriction, through the activation of *α*-adrenoceptors [37], which would obviously be considered as an undesirable action of this substance.

The commercially supplied synephrine produced a very similar inhibitory effect on the mouse uterus to that obtained with HS1. Although the commercial variant appeared slightly more potent than the purified HS1, there was no significant difference in the EC_50_ values, and the slight difference could simply be due to the commercial variant being of higher purity. The effect of HS1 and synephrine on the spontaneous contractions of the mouse uterus was to reduce both their frequency and amplitude, which accounts for the concentration-dependent decrease in the contractile integral. Further support that HS1 is synephrine was provided by the finding that the nonspecific *β*-adrenoceptor antagonist propranolol could block the effect of both these substances. HS1 was also effective in causing relaxation of the mouse uterus, which had been precontracted by KCl-induced depolarisation of the smooth muscle.

N-Methyltyramine has been found to occur in a wide variety of plants [38] including *H. salicornicum* [39, 40]. Like synephrine, N-methyltyramine is a constituent of bitter orange [41]. N-Methyltyramine is thought to act by the same mechanism as tyramine, that is, as an indirect sympathomimetic due to its ability to cause the release of noradrenaline from adrenergic varicosities [42]. This would explain why HS2 also had an inhibitory effect on the spontaneous contractions of the mouse uterus and why propranolol was able to block its inhibitory effect. N-Methyltyramine has previously been shown to increase blood pressure in anesthetized rats and to increase both intensity and rate of contraction of guinea pig right atrium by inducing the release of norepinephrine [43]. Such a mechanism is consistent with what is being proposed here for the effect of N-methyltyramine on the uterus.

Knowing the pharmacology of HS1 and HS2, it might be expected that Bedouin women who are using *H. salicornicum* would experience an increase in their blood pressure; however, this does not appear to be an issue (Dr. Mohamed Fawzy, General Practitioner in the St. Catherine Protected Area, personal communication). One possible reason why this is not a significant problem is that since the crude extract contains other chemical substances, some of these other constituents may counteract the effects of HS1 and HS2. Such a notion is supported by preliminary studies with fraction C, which was found to be capable of causing relaxation of the mouse aorta precontracted with phenylephrine. Thus, the overall pharmacological effect of *H. salicornicum* is going to depend upon what other biologically active substances are present in the plant. 

## 5. Conclusion

This study has provided credible evidence to support the use of *H. salicornicum* by indigenous groups, such as the Bedouin women of Egypt, where conventional medical care is often unavailable and the management of premature birth still remains unsatisfactory [12]. Abnormal contraction of the uterus can lead to premature delivery of the foetus, and it is known to be a significant cause of prenatal morbidity and mortality [44]. The inhibitory action of *H. salicornicum* on uterine contractility, shown in the current study, means that it is likely to be effective as a tocolytic. The active constituents in *H. salicornicum* were found to be synephrine and N-methyltyramine, and they both exert their effects ultimately through the activation of *β*-adrenoceptors on uterine smooth muscle. In summary, *H. salicornicum* is a natural product with biological activity, and this study provides pharmacological evidence supporting the traditional medicinal use of this plant among Bedouin communities for the treatment of perinatal problems.
